# Technology Utilisation and Engagement in Physical Activity of Adolescents With Intellectual Disabilities: A Scoping Review

**DOI:** 10.1111/jar.70193

**Published:** 2026-02-09

**Authors:** Patricia West, Karla Palmer, Brian Abery, Jessica Sender, Alli Walsh, Gwen Wyatt

**Affiliations:** ^1^ College of Nursing Michigan State University East Lansing Michigan USA; ^2^ Institute on Community Integration University of Minnesota Minneapolis Minnesota USA; ^3^ University Libraries, Michigan State University East Lansing Michigan USA

**Keywords:** adolescents, intellectual disability, physical activity, technology

## Abstract

**Background:**

Low levels of physical activity (PA) among adolescents with intellectual disabilities are a serious health concern that increases the risk for chronic health conditions. This scoping review examines technology utilisation that supports PA engagement among adolescents with intellectual disabilities.

**Methods:**

Guided by Arksey and O'Malley's framework, major web‐based searches were conducted. Methodological quality was assessed using the Mixed‐Methods Appraisal Tool.

**Results:**

Forty‐five studies met eligibility. Forty‐four studies utilised technology devices to quantify PA behaviours and address measurement practices. Limited studies utilised technology to influence PA engagement, but suggested the potential value for improving PA participation, duration and intensity to support this population.

**Conclusions:**

Findings highlight the limited utilisation of technology to assist with supporting PA among adolescents with intellectual disabilities. Further, there is a need to emphasise the incorporation of adolescents' perspectives in the development and implementation of future technology‐driven interventions to improve PA engagement.

## Summary

- The majority of studies utilised technology to monitor and measure physical activity behaviour among adolescents with intellectual disabilities.
- Limited studies utilized technology to influence engagement in physical activity among adolescents with intellectual disabilities.
- Evidence suggests exergaming applications as a viable modality for physical activity among adolescents with intellectual disabilities, but capturing the voices of adolescents with intellectual disabilities is needed to support inclusive research
- Future research is needed to access the adoption of technology to address physical activity engagement and health disparities among adolescents with intellectual disabilities.

## 1 Background

The lack of engagement in physical activity (PA) is a leading risk factor for mortality rates globally (World Health Organization 2023) and is defined as any bodily movement that involves energy expenditure (World Health Organization 2023). Approximately 28.9% of youth with intellectual disabilities are obese, compared to 15.5% of youth without intellectual disabilities (Segal et al. 2016). Health disparities experienced by children and adolescents with intellectual disabilities lead to higher rates of risk factors for poorer health than the general population, resulting in an average life expectancy of 20 years less than those without intellectual disabilities (National Institute on Minority Health and Health Disparities 2023; O'Leary et al. 2018). Specifically, among adolescents with intellectual disabilities, the combination of inactivity, potential motor impairments, and accessibility challenges contribute to a higher risk of preventable chronic conditions such as cardiovascular disease, obesity, diabetes, and stroke (Centers for Disease Control and Prevention 2023; McGarty and Melville 2018; Phillips and Holland 2011). These are serious health concerns for adolescents with intellectual disabilities who engage in even less PA than their peers without disabilities, and PA levels continue to decline with aging (CDC 2023; McGarty et al. 2018; Reichard and Stolzle 2011; Rimmer 2022). Studies report prevalence rates for type 2 diabetes among people with intellectual disabilities are estimated to be 2–3 times that of the general population, with expenditures 3.7 times higher than those of diabetics without disabilities (McVilly et al. 2014; MacRae et al. 2015; National Institute on Minority Health and Health Disparities 2023; Reichard and Stolzle 2011).

Adolescents with intellectual disabilities account for about 6% of the 7.3 million public school students in the United States who receive special education or related services under the Individuals with Disabilities Education Act (IDEA; National Center for Education Statistics 2023). Intellectual disability involves significant limitations of both intellectual functioning and adaptive behaviour with an onset before age 22 (American Association on Intellectual and Developmental Disabilities 2023). Behavioural and cognitive challenges experienced by adolescents with intellectual disabilities, in addition to limited access to fitness facilities and competing family responsibilities, increase barriers to participation in PA activities (McGarty et al. 2018; McGarty and Melville 2018). Efforts to influence PA engagement in adolescents with intellectual disabilities require examination to target relevant social determinants of health and multilevel factors impacting health disparities.

Limited population‐specific PA research among adolescents with intellectual disabilities further exacerbates health disparities experienced by this group (McGarty et al. 2018; McGarty and Melville 2018; Van Biesen et al. 2024). Studies often lack valuable details on participants' level of intellectual disabilities, condition/diagnosis and support needs (Borland et al. 2021; Sutherland et al. 2021). Levels of support vary in intensity and frequency and can range from limited or intermittent to extensive for this heterogeneous group. The ability to participate in everyday life and community‐based activity can impact participation in PA (AAIDD 2024). One method found to address access to participation in these activities is innovative digital technology; it can contribute to reducing social gaps and empowering persons with intellectual disabilities, creating alternatives for supporting PA integration in daily living (Tanis and The American Network of Community Options and Resources 2021; Van Biesen et al. 2024).

For the purpose of this review, *technology* refers to a tool, device or equipment utilised to assist with tasks in the environment, such as monitoring exercise adherence or administering exercise (Sulwarajan et al. 2025). Some examples of technology include accelerometers, pedometers, wearable fitness trackers, smartphones, tablet computers and exergaming (such as active video games, virtual reality [VR]). With the speed of technology development and increasing adolescent interest, it is essential to examine technology use and engagement within the context of adolescent health. For example, a systematic review concluded that digital health interventions can potentially be effective for promoting PA among people with intellectual disabilities and/or autism (Van Biesen et al. 2024). Research indicates technology use can have a positive impact on the physiological and psychological benefits of exercise and can increase the likelihood of improving self‐management in adolescents (Charlier et al. 2016; Mesa‐Gresa et al. 2018; Oakes 2022; Qian et al. 2020). Studies promoting self‐management of people with intellectual disabilities emphasise interventions that (a) are tailored to their needs and situations, (b) involve their support network, and (c) transfer skills to daily life (Sandjojo et al. 2018). However, the use of technology tools with demonstrated psychometric properties is limited among adolescents with intellectual disabilities (McGarty and Melville 2018). Research has documented that measurement evidence from the general population may not generalise to samples of individuals with intellectual disabilities (Pitchford et al. 2018b). Thus, there is a need to understand what is known about technology use and engagement in PA among adolescents with intellectual disabilities aged 10–21 years. This specific age group comprises youth in adolescence/late adolescence who are transitioning through stages of physical and psychosocial development, as well as a group eligible for special education and related services under IDEA Part B (U.S. Department of Education 2023; World Health Organization 2025).

Understanding technology utilisation for PA engagement among adolescents with intellectual disabilities may provide insights on how to optimally approach the development of a multimodal intervention to increase PA, monitor technology limitations and reduce barriers to address health disparities during a critical developmental period. Therefore, the purpose of this scoping review is to examine what types of technology (e.g., smartphones, tablet computers, VR, augmented reality, exergames, videogames, fitness wearables/trackers, etc.) have been utilised and assessed (e.g., barriers/facilitators) among adolescents with intellectual disabilities, and to evaluate what is known about PA engagement in this population. This review depicts a novel approach to understanding technology that has been employed in research surrounding PA in adolescents with ID and its use to facilitate engagement in PA. Additionally, this review examines the quality of research methodology utilised in previous studies to identify areas for additional research to improve health outcomes in this population.

## 2 Methods

A scoping review is an iterative process, requiring researchers to search, examine, synthesise, and identify gaps in the existing literature (Arksey and O'Malley 2005). This methodological framework allows for the inclusion of diverse study designs in a single assessment of the literature to comprehensively understand the overall state of research activity in a particular area of inquiry (Arksey and O'Malley 2005; Tricco et al. 2018). This approach is well‐suited to evaluating the state of the science to identify knowledge gaps regarding technology utilisation and engagement in PA among adolescents with intellectual disabilities, which may help in the development of innovative interventions to reduce health disparities. In addition, the Arksey and O'Malley (2005) framework includes five main stages: (a) developing the research question(s) and eligibility criteria; (b) identifying relevant studies; (c) selection of studies; (d) charting the data; and (e) collating, summarising, and reporting the results.

**FIGURE 1:**
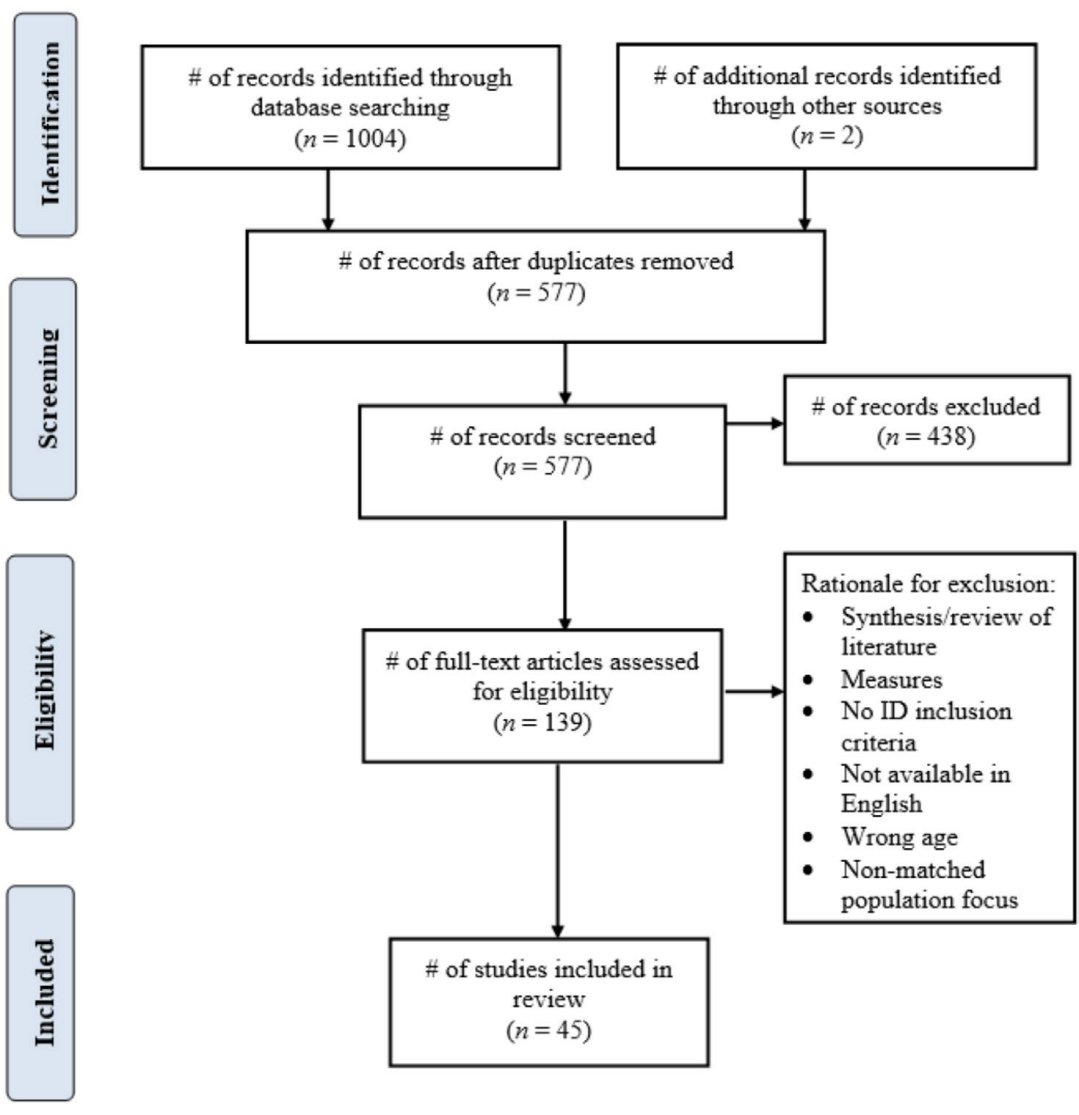
PRISMA diagram. Flow diagram of the retrieval and screening process.

The Preferred Reporting Items for Systematic Reviews and Meta‐Analysis (PRISMA) (see Figure 1; Liberati et al. 2009) was used to report the process of obtaining and including literature for this review. The PRISMA Extension for Scoping Reviews (PRISMA‐ScR) checklist was utilised to enhance the rigour and reporting of the review (Tricco et al. 2018). Given the varied methodological approaches incorporated into this review, the Mixed Methods Appraisal Tool (MMAT) was employed for quality appraisal of the studies (Hong et al. 2018). For transparency, the study was registered via the Open Science Framework (OSF).

### 2.1 Stage 1. Research Questions

#### 2.1.1 Research Questions

The review was guided by the following research questions:

- What types of technology have been used to increase engagement in PA among adolescents aged 10–21 years with intellectual disabilities?
- What types of technology have been used to measure PA among members of the target population, adolescents aged 10–21 years with intellectual disabilities, and to what degree have these measures demonstrated adequate psychometric characteristics?
- What are the primary barriers and facilitators of utilising technology to stimulate PA among adolescents aged 10–21 years with intellectual disabilities?
- What evidence currently exists that indicates a positive/negative impact in the use of technology for engagement in PA of adolescents aged 10–21 years with intellectual disabilities?

#### 2.1.2 Inclusion Criteria

Studies that were included met the following criteria: (a) individuals in adolescence aged 10–21 years with intellectual disabilities who participated in PA without restrictions; (b) intellectual disability condition at all levels (e.g., mild, moderate, severe) known in the sample demographics; (c) technology utilised to capture outcomes or encourage PA; (d) findings related to PA; (e) published in English; (f) any study design; (g) peer reviewed; and (h) unrestricted publication dates.

#### 2.1.3 Exclusion Criteria

Excluded studies were those with (a) adolescents aged 10–21 years diagnosed with an acquired brain injury; (b) those that did not designate intellectual disability conditions in sample demographics; and (c) participants who were under 10 and over 21 years of age. Publications with mixed samples (i.e., multiple age ranges, mixed with other developmental disabilities) were excluded if the study did not clearly differentiate the results for the cohort of individuals with intellectual disabilities and/or the specific age range. Systematic reviews, meta‐analyses, reviews of the literature, unpublished studies/documents and commentaries were excluded.

### 2.2 Stage 2. Identifying Relevant Studies

The first author consulted with the College of Nursing librarian to conduct an initial literature search on March 28, 2023. The search was run in the following databases: CINAHL, PubMed, Embase, PsycINFO and SportDiscus. Controlled vocabulary (Medical Subject Headings [Mesh] and CINAHL subject headings) and appropriate and relevant keywords were utilised. The search was modified for each database to include controlled vocabulary specific to the database when appropriate, but keywords remained the same across databases. The search focused on intellectual, developmental or cognitive disorders; adolescent or teen population; PA; and technology. Each of these broad categories had many keywords and subject headings associated with them. No date limits were applied. The search was rerun on August 2, 2023, and then again on March 6, 2025, to expand the search to include MeSH headings for both developmental and intellectual disabilities, as well as to screen for updated published work. The third search identified an additional 190 citations for review, and five studies were included (see Figure 1). Additionally, reference lists of pertinent reviews were manually screened for additional articles. A full search strategy for each database can be found in Appendix A.

### 2.3 Stage 3. Selection of Studies

Abstracts and full‐text articles were independently screened by the primary and secondary authors utilising Covidence (2023; https://www.covidence.org/), a web‐based software platform. OneDrive (https://www.microsoft.com/en‐us/microsoft‐365/onedrive/online‐cloud‐storage) was used to manage, store, and share selected articles. If the relevance of the study was unclear, as was the case with two studies, the third author was consulted until a mutual consensus between the three researchers was reached for included or excluded studies.

### 2.4 Stage 4. Charting Data

A descriptive‐analytical method for data extraction was utilised to collect standard information from each study (Arksey and O'Malley 2005). Information collected from each study was managed in Microsoft Excel to provide a summary of the literature. Data extracted from the studies included author, year of publication, country, methodology, purpose, sample characteristics, key findings, instruments/measures, physiological measures, type of technology, intervention and/or engagement components, type of PA, and intensity of PA.

#### 2.4.1 Quality Appraisal

According to Arksey and O'Malley (2005), quality of evidence is generally not the focus of scoping reviews. However, quality should be addressed in a meaningful way. The MMAT is designed for the appraisal of qualitative, quantitative, and mixed‐methods studies (Hong et al. 2018). Quality was scored by answering five criteria questions for each methodological category with fewer criteria met correlating to a lower quality score. In Table 1, studies that met one criterion had a score of 20% (*), while studies that met all criteria had a score of 100% (*****).

**TABLE 1:** Description of included studies (*N* = 45).

Author, year, country	Method/design	Purpose	Participants	MMAT
Beets et al. (2007), US	Quant Descriptive	To examine the accuracy of pedometer measured steps and time in a population of youth diagnosed with developmental disabilities	18 elementary and middle schoolers; 7 boys (*M* = 10.0 years, SD = 3.4 years); 11 girls (*M* = 9.7 years, SD = 3.1 years); Diagnosed with ASD (*n* = 3), mild mental retardation (17), DS (3), visual impaired (1), seizure disorder (1), and juvenile arthritis (1).	***
Bellamy et al. (2020), Australia	Quant Prospective cohort (single arm)	To explore the feasibility of a 16‐week school‐based group exercise program to increase participation in MVPA and reduce cardio‐metabolic risk among children with moderate‐to‐severe ID	10 children (*M* = 10.7 years, range: 9.4–12.6 years); 8 boys; 2 girls; Diagnosed with moderate ID (*n* = 4); severe/profound ID (*n* = 6); including ASD, DS, and Sotos syndrome.	****
Boddy et al. (2015), UK	Quant Cross‐sectional	To investigate levels of habitual PA and recess play behaviour in a group of children and young people with ID and examine whether there were any differences by sex, age group, and ID group	33 youth; aged 5–15 years. (*M* = 9.97 years.; SD 0.3 years); 26 boys; 7 girls; mix of diagnosed and not diagnosed with ASD.	***
Carrogi‐Vianna et al. (2017), Brazil	Quant Cross‐sectional	To evaluate movement acceleration characteristics in adolescents DS and TD, while playing bowling and golf videogames on the Nintendo Wii.	54 adolescents; 21 with DS, 7 girls (*M* = 11.2 years), 14 boys (*M* = 11.9 years); 33 TD, 16 girls (*M* = 11.8 years), 17 boys (*M* = 12 years).	***
Downs et al. (2016), UK	Quant Cross‐sectional	To objectively investigate habitual PA and sedentary behaviours in children and adolescents with ID and to examine the tempo of PA by sex, age, and disability.	38 children and adolescents; aged 5–15 years (*M*= 9.97); 29 boys; 9 girls; Diagnosed with MLD (*n* = 19), SLD (*n* = 51), SLD and ASC (*n* = 27); Additional conditions and included DS, global developmental delay, microcephaly, CP, ADHD, Angleman syndrome, dyspraxia, and visual impairment.	***
Einarsson et al. (2016), Iceland	Quant Cross‐sectional	To investigate PA and sedentary time during school hours vs. after school hours among Icelandic school children with ID and to compare them to an age‐ and sex‐matched group of TDI children, as well as to investigate any potential sex differences in PA and sedentary time	184 school children; 93 TDI (*M* = 11.9 years, SD = 2.7); 91 with ID (*M* = 11.9 years, SD = 2.9); Diagnosed with moderate‐to‐severe ID, mild‐to‐moderate ID, cerebral palsy (*n* = 3), and epilepsy (*n* = 4).	***
Einarsson et al. (2015), Iceland	Quant Cross‐sectional	To investigate PA and PA patterns with accelerometers among Icelandic school children with ID and to compare them with an age‐ and sex‐matched group of TDI children.	184 school children; 93 TDI (*M* = 11.9 years, SD = 2.7), 58 boys (*M* = 11.9 years, SD = 2.9), 35 girls (*M* = 12 years, SD 2.3); 91 with ID (*M* = 11.9 years, SD = 2.9), 62 boys (*M* = 11.8 years, SD = 2.8), 29 girls (*M* = 11.9 years, SD = 2.6); Diagnosed with moderate–severe ID, mild–moderate ID, cerebral palsy (*n* = 3), epilepsy (*n* = 4).	***
Esposito et al. (2012), US	Quant Cross‐sectional	To examine the PA patterns of children with DS.	104 children (*M* = 11.81 years, SD = 2.21); 57 boys, 47 girls; 25 aged 8–9.9 years (*M* = 9.26 years, SD = 0.48); 38 aged 10–11.9 years (*M* = 10.93 years, SD = 0.68); 27 aged 12–13.9 years (*M* = 13.03 years, SD = 0.50); 14 aged 14–15.9 years (*M* = 15.01 years, SD = 0.56); Diagnosed with DS. 85.6% Caucasian, 4.8% African American, 1.9% Asian American, 1% Hispanic or Latin American descent, 4.8% other.	***
Fleming et al. (2023), US	Quant RCT	Compare PA between (1) participants randomised to receive the 6‐month FBBI and those who were in the concurrent waitlist control group, and (2) these same participants randomised to receive the maintenance intervention (FBBI‐M) and those in a no further treatment control group (FBBI‐C).	21 participants, 100% White; *FBBI & FBBI‐M: n* = 4 (*M* = 17.7 years, SD = 3.0), 1 boys, 3 girls, 25% Hispanic/Latino; With DS (*n* = 3), other IDD: (*n* = 1); *Waitlist FBBI & FBBI‐C*: *n* = 5 (*M* = 20.4 years, SD = 1.4), 2 boys, 3 girls, 0% Hispanic/Latino; With DS (*n* = 2), other IDD: (*n* = 3); *Intermediate FBBI & FBBI‐M: n* = 6 (*M* = 16.4 years, SD = 1.2), 2 boys, 4 girls, 0% Hispanic/Latino; With DS (*n* = 4), other IDD (*n* = 2); *Immediate FBBI & FBBI‐C*: *n* = 6 (*M* = 18.0 years, SD = 2.2), 2 boys, 4 girls, 0% Hispanic/Latino; With DS (*n* = 3), other IDD (*n* = 3)	***
Gobbi et al. (2018), Italy	Mixed Methods Triangulation	(1) To compare levels of PA, enjoyment of PA and perceived exertion by students with intellectual disabilities in a PTPE versus SPE, and (2) to explore if participants showing overweight or normal weight benefited differently from the two conditions.	19 students; 15 boys (*M* = 17.4, SD = 1.7); 12 normal weights; 7 overweight (using WHO growth reference); Diagnosed with mild‐to‐moderate ID level, excluded DS and ASD.	****
Hao and Razman (2023), China	Quant Cross‐sectional	To investigate the MVPA levels of children with ID during PE classes in China and the differences in MVPA levels according to gender and grade.	53 children; 35 boys (*M* = 12.35, SD = 1.64), 18 girls (*M* = 13.11, SD = 1.61); diagnosed with severe ID with IQ of 25–39.	***
Izquierdo‐Gomez et al. (2017), Spain	Quant Longitudinal observational study	To examine 1 and 2‐year longitudinal changes in objectively measured PA among a relatively large sample of adolescents with DS.	99 adolescents at baseline (2011–2012); 38 girls (*M* = 15.6 years, SD = 2.2), 61 boys (*M* = 16.1 years, SD = 2.5); 92 adolescents at follow up (2012–2013); 36 girls (*M* = 16.1 years, SD = 2.2); 56 boys (*M* = 17.1 years, SD = 2.5); 88 at 2nd follow up (2013–2014); 32 girls (*M* = 17.5 years, SD = 2.1); 56 boys (*M* = 18.1 years, SD = 2.5); diagnosed with DS.	***
Izquierdo‐Gomez et al. (2015), Spain	Quant Cross‐sectional	To identify correlates of objectively measured PA in a relatively and heterogeneous sample of adolescents with DS.	98 adolescents; 63 boys; 35 girls; 47 aged ≤ 15; 51 aged ≥ 16; diagnosed with DS.	****
Lobenius‐Palmér et al. (2018), Sweden	Quant Cross‐sectional	To investigate habitual PA, sedentary time, and meeting PA recommendations across several types of disabilities and in comparison, with youth with TD.	102 youth; 59 boys; 43 girls; 16 with physical/visual impairment (*M* = 14 years, SD = 3.6), girls: *M* = 12.9 years, SD = 2.6, boys: *M* = 15.4 years, SD = 4.3; 42 with ID (*M* = 14.7 years, SD = 3.7), girls: *M* = 16.8 years, SD = 2.8, boys: *M* = 12.9 years, SD = 3.4; 25 with ASD (*M* = 14.0 years, SD = 3.6), girls: *M* = 14.3 years, SD = 3.8, boys: *M* = 14.0 years, SD = 3.7; 19 with HI/deafness (*M* = 11.1 years, SD = 3.0), girls: *M* = 12.9 years, SD = 2.3, boys: *M* = 9.8 years, SD = 2.8	****
Matute‐Llorente et al. (2017), Spain	Quant Cross‐sectional	To investigate whether PA levels are related to the femoral neck bone mass distribution in a sample of males and females with DS aged 12–18 years.	26 participants; 12 girls (*M* = 15.7 years, SD = 2.9); 14 boys (*M* = 16.5 years, SD = 2.6); diagnosed with DS.	***
Matute‐Llorente et al. (2013a), Spain	Quant Cross‐sectional	(1) To determine if adolescents with DS accomplish the PA guidelines for health objectively assessed comparing their PA patterns with adolescents without disabilities; (2) To test the relationship between PA levels and cardiovascular fitness in adolescents with DS.	42 adolescents; 15 in control group, (*M* = 13.6 years, SD = 3.0). 8 girls (*M* = 13.3 years, SD = 3.1), 7 boys (*M* = 14.0 years, SD = 3.1); 27 with DS (*M* = 16.2 years, SD = 2.9), 13 girls (*M* = 15.8 years, SD = 3.2), 14 boys (*M* = 16.5 years, SD = 2.7).	***
Matute‐Llorente et al. (2013b), Spain	Quant Cross‐sectional	(1) To describe PA patterns in adolescents with DS compared to their counterparts and (2) to determine relationships between PA and risk of having low bone mass in adolescents with DS.	20 adolescents with DS (*M* = 14.7 years, SD = 2.2). 10 boys, 10 girls; 20 without disability (*M* = 13.2 years, SD = 2.8), 10 boys, 10 girls.	***
McMahon et al. (2020), US	Mixed Methods Nonrandomized intervention study	To determine whether VR exergaming can influence the PA levels in youth with IDD.	4 youth; a 14‐year‐old female student diagnosed with fetal alcohol syndrome; a 17‐year‐old male student diagnosed with ASD; a 16‐year‐old male diagnosed with DS; a 21‐year‐old male student diagnosed with ID and other health impairments (i.e., breathing, feeding).	*****
Pan et al. (2015), Taiwan	Quant Cross‐sectional	To compare PA levels during physical education and recess in adolescents with ID (inclusive vs. self‐contained classrooms) and typically developing adolescents.	80 adolescents; 40 TD (*M* = 14.33 years, SD = 1.06), 10 girls, 30 boys; 40 with ID. 10 girls, 30 boys, 20 from inclusive classrooms (*M* = 14.84 years, SD = 0.95), 20 from self‐contained classrooms (*M* = 14.25 years, SD = 1.07), diagnosed with ID: slight (*n* = 21), medium (*n* = 14), high (*n* = 3) and total (*n* = 2), Secondary diagnosis: HI (*n* = 2), speech and language disorder (*n* = 2).	***
Peiris et al. (2017), Australia	Quant Descriptive	To assess the reliability and validity of the SenseWear armband and RT3 activity monitor in estimating energy expenditure of young people with DS.	10 young people (*M* = 20.0 years, SD = 2.0; range 16–24 years); 5 girls, 5 boys; 2 aged 10 to < 18 years, 8 young adults; 4 normal weight, 2 overweight, 4 obese; Diagnosed with DS; other conditions PDA (*n* = 3), mitral valve regurgitation (*n* = 1); ID class (parent report): Mild (*n* = 5), Moderate (*n* = 5).	***
Phillips and Holland (2011), UK	Quant Cross‐ sectional	To investigate, using accelerometers, the levels of PA undertaken by a large sample of individuals with ID, to estimate the percentage of participants meeting physical activity recommendations, and to study any association with age, gender, and level of ID.	152 individuals, 74 boys, 78 girls; 7 aged 12–15 (3 boys, 4 girls); 75 aged 16–34 (37 boys, 38 girls); 31 aged 35–44 (17 male, 14 female); 18 aged 45–54 (9 male, 9 female); 21 aged 55–64 (8 male, 13 female); Diagnosed with idiopathic ID (*n* = 61), DS (*n* = 79), ASD (*n* = 9), Russell‐Silver syndrome (*n* = 1), Treacher Collins syndrome (*n* = 1), Beckwith‐Wiedemann syndrome (*n* = 1); level of ID of total sample, mild: Males (33.8%), Females (38.0%), moderate: Males (39.4%), Females (33.8%); Severe: Males (26.8%), Females (33.8%).	***
Pincus et al. (2019), US	Quant Non‐randomised intervention study	To replicate and extend the work of Hustyi et al. (2012) by determining the effects of different environmental contexts on levels of PA displayed by adolescents with IDD, and to subsequently determine individual preference for sedentary activities and activities that may evoke MVPA	3 adolescents; “Melanie,” a 16‐year‐old female previously diagnosed with pica, disruptive behaviour disorder—not otherwise specified (DBD‐NOS), ASD, severe ID, and ADHD, BMI class normal; “Andrew,” an 18‐year‐old male previously diagnosed with rumination disorder, DBD‐NOS, anxiety disorder‐NOS, ASD, and ID‐level unspecified. BMI class normal; “Christina,” a 17‐year‐old female previously diagnosed with DBD‐NOS, stereotypic movement disorder with self‐injurious behaviour, obsessive compulsive disorder, ADHD, DS, ASD, and moderate ID. BMI class obese.	***
Pitchford et al. (2018a), US	Quant Cross‐sectional	To measure adiposity using an advanced measurement technique (DXA) capable of examining differences between adolescents with and without DS and the subsequent associations with PA behaviour.	39 adolescents; 22 with DS (*M* = 14.96 years, SD = 1.92), 8 girls, 14 boys, 90% Caucasian, 5% African American, and 5% Hispanic; 17 TD (*M* = 15.08 years, SD = 2.12), 10 girls, 7 boys, 100% Caucasian.	***
Pitetti et al. (2009), US	Quant Cross‐sectional	To examine the accuracy of pedometry in children and adolescents with ID engaged in dynamic movements during adapted physical education.	24 children and adolescents with mild ID; 11 boys (*M* = 14.7 years, SD = 2.7); 13 girls (*M* = 13.1 years, SD = 3.2) 18 white non‐Hispanic, 6 Hispanic.	***
Ptomey et al. (2024), US	Quant RCT	Examine the results from an 18‐month trial in adolescents with IDD, which compared accelerometer‐assessed daily MVPA between participants randomised to a home‐based, remotely delivered group exercise intervention delivered to adolescents only (AO) or the same home‐based group exercise intervention plus education/support sessions delivered to both the adolescent and a parent (A + *P*).	116 adolescents mild to moderate ID; *AO arm: n* = 59 (*M* = 15.4, SD = 3.1), 31 boys, 28 girls, 72.9% White, 10.2% Black, 16.9% Other, 6.8% Hispanic/Latino, With DS (*n* = 32), ASD (*n* = 15), other IDD: (*n* = 14); *A + P arm*: *n* = 57 (*M* = 15.6, SD = 2.9), 22 boys, 35 girls, 78.9% White, 7% Black, 6.8%, 14% Other, 8.8% Hispanic/Latino, With DS (*n* = 32), ASD (*n* = 14), other IDD: (*n* = 12)	*****
Ptomey et al. (2022a), US	Quant secondary data analysis from PA trial	This study examined intrapersonal, interpersonal, and environmental factors and their association with MVPA and sedentary time in adolescents with IDD.	92 adolescents; aged 10–21 (*M* = 15.5 years, SD = 3.0); 40 boys, 52 girls; diagnosed with moderate IDD (*n* = 31), specifically DS (*n* = 49), ASD (*n* = 22), other IDD (*n* = 18).	***
Ptomey et al. (2022), US	Quant RCT (trad: 3 arms)	To examine changes in light, MVPA and sedentary time, and the association between changes in MVPA and weight loss in adolescents and young adults with IDD and overweight and obesity participating in a 6‐month multi‐component weight loss intervention.	110 participants mild to moderate IDD; *Face to face & conventional diet: n* = 36 (*M* = 16.3 years, SD = 2.7), 20 boys, 16 girls, 83% White; 94% Non‐Hispanic/Latino; With DS (*n* = 17), ASD (*n* = 5), other IDD: (*n* = 11); *Remote delivery & conventional diet*: *n* = 39 (*M* = 15.6 years, SD = 1.7), 15 boys, 24 girls, 97% White, 95% Non‐Hispanic/Latino, With DS (*n* = 21), ASD (*n* = 14), other IDD: *n* = 4; *Remote delivery & Enhanced stop light diet: n* = 35 (*M* = 16.7 years, SD = 2.8), 17 boys, 16 girls, 83% While, 89% Non‐Hispanic/Latino; With DS (*n* = 15), ASD (*n* = 13), other IDD (*n* = 7).	*
Queralt et al. (2016), Spain	Quant Descriptive	To examine daily PA patterns (weekdays and weekend days) of adolescents with IDs among boys and girls. In addition, the contributions of PA at school (including school recesses and physical education time) and PA outside of school were considered in our analysis.	35 adolescents with mild to moderate ID aged 12–20 years (*M* = 15.3 years SD = 2.7); 22 boys (*M* = 14.7 years, SD = 2.6); 13 girls (*M* = 16.2 years, SD = 2.7); Diagnosed with DS (*n* = 9).	***
Ryuh et al. (2022), US	Quant Non‐randomised intervention study	To examine physiological responses (measured as heart rate, rate of perceived exertion) and psychological response (measured as PA enjoyment scale) in young adults with ID, and to assess the feasibility of exergaming.	7 young adults aged 19–22 years (*M* = 20.3 years, SD = 1.1); 4 boys; 3 girls; Diagnosed with mild‐to‐moderate IDD; IQ range: 50–89 (*M* = 68).	****
Shields et al. (2017a), Ireland	Quant Exploratory cohort study	To examine the relationship between (1) PA and body size, (2) cardiovascular fitness and body size and (3) PA and cardiovascular fitness in children with DS.	14 children aged 7–17 years (*M* = 12.9 years, SD = 3.5); 8 boys (*M* = 11.8 years, SD = 3.9); 6 girls (*M* = 14.5 years, SD = 2.6); Diagnosed with DS, including mild ID (*n* = 3), mild‐to‐moderate ID (*n* = 8), mod ID (*n* = 3).	***
Shields et al. (2017b), Australia	Quant Prospective cohort study	To investigate the relationship between foot structure, footwear fit, and levels of PA in children and adolescents with DS.	20 children and adolescents aged 5–18 years (*M* = 11.2 years, SD = 3.8); 12 boys; 8 girls; Diagnosed with DS, including mild ID (*n* = 7), mod ID (*n* = 6), severe ID (*n* = 2), and unknown ID (*n* = 5).	***
Shields et al. (2009), Australia	Quant Descriptive	To measure the usual amount and intensity of PA children with DS complete over 7 days to determine if they satisfied published guidelines on the amount of PA children should undertake daily (60 min of MVPA/day and 20 min of continuous vigorous activity at least 3 times/week).	23 children aged 7–17 years (*M* = 12.1 years, SD = 3.1); 16 boys; 7 girls; Diagnosed with DS, including mild ID (*n* = 9), mild‐to‐moderate ID (*n* = 3), mod ID (*n* = 11).	***
Shih (2011), Taiwan	Quant Time series (A‐B‐A‐B design)	To evaluate whether two individuals with ID would be able to actively perform simple physical activities—walking to the correct location and standing there—by controlling their favourite stimulation using Wii Balance Boards with a newly developed standing location detection program	2 individuals; 18 years; ID: profound, obese; 17 years; ID: moderate; limited verbal ability.	**
Shih et al. (2011), Taiwan	Quant Time series (A‐B‐A‐B design)	To assess whether participants would be able to actively follow simple instructions to perform designated PA with simple instructions.	2 individuals; one boy,18 years; ID: profound, non‐verbal; one girl, 17 years; ID: middle; normal verbal ability.	**
Sit et al. (2017), China	Quant Cross‐ sectional	To use accelerometry to objectively assess the PA and ST of boys and girls with different disability types in school PA settings; to determine the relative contributions of the accrual of PA and ST in these settings to overall school MVPA and ST in children with disabilities.	259 children aged 6–21 years (*M* = 13.04 years, SD = 4.45); ID (mild): *n* = 92, 55 boys, 37 girls; ID (mod): *n* = 59, 36 boys, 23 girls; ID (severe): *n* = 35, 18 boys, 17 girls; Diagnosed with, visual impairment, hearing impairment, and social development problems.	*****
Stanish et al. (2019), US	Quant Case control Non‐randomised	To examine and compare time spent in objectively measured MVPA and the types and frequency of physical activities performed by youth with and without ID. Hypotheses that youth with ID would spend less time in MVPA compared to TD youth, and that they would participate in fewer distinct physical activities, and at lower frequencies.	38 youth with ID (*M* = 16.8 years, SD = 1.9), 17 boys, 21 girls, 45% White; 60 youth without ID (*M* = 16.8 years, SD = 1.9), 36 boys, 24 girls, 60% White.	*****
Sundahl et al. (2016), Sweden	Quant Case–control Non‐randomised	To investigate the number of steps taken among adolescent and young adult women and men with ID compared to age‐matched control groups without ID, and to examine whether number of steps taken was associated with the BMI.	52 young adults with mild to moderate ID aged 16–20 years, 25 boys (*M* = 18.4 years, SD = 1.0), 27 girls (*M* = 18.0 years, SD = 1.3); 48 young adults without ID aged 17–20 years, 27 boys (*M* = 18.0 years, SD = 0.9); 21 girls (*M* = 17.9 years, SD = 0.9).	****
Wang et al. (2022), China	Quant Cross‐sectional	(1) To assess whether there are associations between FMS and MVPA levels of children and adolescents with ID and (2) to analyse whether there are gender and age differences in such an association. We hypothesize that (1) there is a significant association between FMS and MVPA levels of children and adolescents with ID and (2) the association between FMS and MVPA is moderated by gender and age.	93 children and adolescents aged 8–17 years (*M* = 13.27 years, SD = 3.35), 63 boys, 30 girls; Utilised the WHO classification of age groups; 7–12 years (*n* = 49), 34 boys, 15 girls; 13–17 years (*n* = 44), 29 boys, 15 girls.	*****
Wouters et al. (2019), Netherlands	Quant Cross‐sectional	(a) What is the volume and intensity of PA of children and adolescents with moderate‐to‐severe ID?; (b) How many participants are active enough to reach the PA recommendations of 60‐min MVPA per day?; and (c) Which child characteristics (age, sex, level of ID, DS, motor development) are associated with PA outcomes?	68 children and adolescents (*M* = 9.4 years, SD = 4.3); 43 boys, 25 girls; 28 aged 2–7 years; 20 aged 8–12 years; 20 aged 13–18 years; Diagnosed with DS (*n* = 16), ASD (*n* = 20), Mod ID (*n* = 30), Severe ID (*n* = 38).	****
Xanthopoulos et al. (2023), US	Quantitative secondary data analysis from cross‐sectional	(1) to compare minutes and types of PA and SA (sedentary activity) in youth with and without DS, (2) to examine the relationships between PA and its traditional risk factors [age, sex, race, and BMIZ] in the groups, and (3) to explore the relationship of PA with visceral fat in both groups.	77 youth with DS (*M* = 15.1 years, SD = 3.4) 49 girls, 28 boys, 83% White, 14% African American, and 3% other; 57 youth without DS (*M* = 15.0 years, SD = 3.3), 29 girls, 28 boys, 77% White, 19% African American, and 4% other.	***
Xu and Wang (2023), China	Quant Cross‐sectional	To (1) investigate the prevalence of Chinese children and adolescents with ID that meet the 24‐h movement guidelines (in isolation or combination) and (2) identify the socio‐demographic correlates of meeting the 24‐h movement.	319 children and adolescents (*M* = 12.93 years, SD = 3.32), 198 boys, 121 girls; Diagnosed with mild ID (*n* = 39), mod ID (*n* = 84), severe ID (*n* = 150), and profound ID (*n* = 46).	****
Yang et al. (2024), South Korea	Quant Cross‐sectional	To assess PA levels in children and adolescents with DS using an activity tracker (Fitbit) and a parental proxy questionnaire to (1) compare the PA levels of a group of school‐age children with DS to those of adolescents with DS, (2) differentiate between the PA levels on weekdays and weekends, and (3) assess the agreement with the parental proxy questionnaires.	32 children and adolescents; School‐aged children (Mdn = 9.00 years), adolescents (Mdn = 13.25 years); 17 boys, 15 girls; Diagnosed with DS	****
Yang et al. (2022), China	Quant Cross‐sectional	To (1) examine the associations between PA levels, QOL, and self‐concept (SC); (2) investigate the underlying mechanisms, such as mediation and interaction effects on the associations among PA levels, QoL, and SC; and (3) examine the moderating effects of personal and environmental factors on PA levels, QoL, and SC in children and adolescents with ID during the COVID‐19 pandemic.	117 children and adolescents (*M* = 13.17 years, SD = 3.82); 86 boys, 31 girls; Diagnosed with mild ID (*n* = 61) and mod ID (*n* = 56).	***
Zhong et al. (2025), China	Quant Cross‐sectional	To examine the associations between MVPA and EBPs in Chinese children and adolescents with ID.	116 children and adolescents (*M* = 13.11 years, SD = 3.05); 75 boys, 41 girls; Diagnosed with mod ID (*n* = 36), severe ID (*n* = 80).	***
Zhu et al. (2020), China	Quant Cross‐sectional	To examine reactivity to accelerometer measurement among youth with moderate and severe ID.	175 youth (*M* = 13.11 years, SD = 2.9); 131 boys, 44 girls, Ethnic: 100% Han; Diagnosed with mod ID (*n* = 108) and severe ID (*n* = 67).	***

*Note:* ADHD = Attention‐deficit/hyperactivity disorder; Adolescent only = AO; Adolescent + parent = AO + *P*; ASD = Autism spectrum disorder; ASC = Autism spectrum condition; BMI = Body mass index; BMIZ = Body mass index z‐score; CP = Cerebral palsy; DBD‐NOS = Disruptive behaviour disorder not otherwise specified; DS = Down syndrome; DXA = Dual‐energy x‐ray absorptiometry; EBPs = Emotional and behaviour problems; FBBI = Family‐based behavioural intervention; FBBI‐C = No‐maintenance control; FBBI‐*M* = Maintenance intervention; FMS = Fundamental movement skill; HI = Hearing impairment; ID = Intellectual disabilities; IDD = Intellectual and developmental disabilities; IQ = Intelligence quotient; MLD = Moderate learning disabilities; MMAT = Mixed methods appraisal tool; MVPA = moderate‐to‐vigorous‐intensity physical activity; PA = Physical activity; PDA = Patent ductus arteriosus; PE = Physical education; PTPE = Peer‐tutored physical education; QOL = Quality of life; SC = Self‐concept; SLD = Severe learning disabilities; ST = sedentary time; SPE = School physical education; TD = Typical development; TDI = Typical developed individual; VR = Virtual reality. * Studies that had met one MMAT criterion had a score of 20%, **Two criterion met had a score of 40%, ***Three criterion met had a score of 60%, ****Four criterion met had a score of 80%, and *****Studies meeting all 5 criterion had a score of 100%.

## 3 Results

### 3.1 Stage 5. Collating, Summarising, and Reporting the Results

After initial searching and the two updated searches, there were 1004 total records. After duplicate removal, 577 records remained for title and abstract review. After title and abstract review, full text review was conducted, and 139 articles were retrieved. A total of 45 records met the full eligibility criteria and were therefore included in the scoping review.

#### 3.1.1 Quality Appraisal

Two reviewers independently evaluated and categorised the 45 included studies with the MMAT criteria according to their study design (see Table 1). Discrepancies between reviewers were resolved through discussion. Inter‐rater reliability was evaluated following two independent evaluations. Across 45 studies, a total of 21 disagreements among five MMAT criteria categories were identified, equating to 90.7% agreement across quality scores. The MMAT categories were based on the study design; however, no qualitative studies met the review criteria. Three mixed‐methods studies were included in the review. The MMAT also evaluated the source and type of data, sampling, instrumentation and data analysis of findings (Hong et al. 2018).

Overall, the MMAT quality assessment scores for quantitative studies ranged from meeting one criterion to all five criteria (see Table 1). Some issues identified in the quantitative studies related to the justification of the sample strategy and procedure, as well as risk of nonresponse bias (*n* = 36). Of the three randomised control trial studies (RCT; Fleming et al. 2023; Ptomey et al. 2024; Ptomey, Lee, et al. 2022b), issues were noted with randomization of participants (Ptomey, Lee, et al. 2022b), whether the outcome assessors were blinded to the intervention provided (Fleming et al. 2023; Ptomey, Lee, et al. 2022b), and participant adherence to the assigned intervention (Fleming et al. 2023; Ptomey, Lee, et al. 2022b). The MMAT quality assessment scores for all three mixed‐methods studies ranged from meeting four to five criteria (Gobbi et al. 2018; McMahon et al. 2020; Stanish et al. 2019). Issues identified related to the data collection methods (Gobbi et al. 2018).

#### 3.1.2 Overview of the Literature

The 45 studies included in this review were published between 2007 and 2025, with increasing patterns of publication over time; 87% of studies (*n* = 39) were published between a 10‐year period from 2013 to 2025. Studies were conducted in the United States (*n* = 12); Spain and China (*n* = 7); Australia (*n* = 5); Taiwan and the United Kingdom (*n* = 3). Sweden and Iceland (*n* = 2), Brazil, Italy, South Korea, and the Netherlands each published one. Most studies were non‐intervention studies (*n* = 33). Nine were intervention studies and three mixed methods (Tables 1 and 2). The 45 studies included participant sample sizes ranging from two to 319, with ages ranging from 10 to 21 years. In 98% (*n* = 44) of the studies, samples included both male and female adolescents. One study did not report participants' sex (Shih 2011). Race was reported in 16% (*n* = 7) of publications in which participants identified primarily as Caucasian (Esposito et al. 2012; Pitchford, Adkins, et al. 2018a; Pitetti et al. 2009; Ptomey et al. 2024; Ptomey, Lee, et al. 2022b; Stanish et al. 2019; Xanthopoulos et al. 2023).

**TABLE 2:** Summary characteristics of studies (*N* = 45).

Characteristics	# of studies
Year	
2025–2019	19
2018–2013	20
2012–2007	6
Study design	
Quantitative RCT	3
Quantitative non‐randomised	9
Quantitative descriptive	30
Mixed methods	3
Qualitative	0
Location	
US	12
Spain, China	7
Australia	5
Taiwan	3
UK	3
Iceland, Sweden	2
Brazil, Italy, Netherlands, South Korea	1
Reporting of intellectual disability	
Medical provider	17
School	10
Parent	10
State center/department	4
Researcher	4
Technology (not mutually exclusive)	
Accelerometer	35
Pedometer	5
NintendoWii	4
Apple watch	1
Eee Box minicomputer	1
Fitbit Charge	3
iPad	4
Oxycon mobile	1
VR	1
Xbox 360	1

Abbreviations: RCT = Randomised control trial; UK = United Kingdom; US = United States; VR = Virtual reality.

The majority (73.3%) of the 33 non‐intervention studies were cross‐sectional; only one was longitudinal (Izquierdo‐Gomez et al. 2017; see Table 1). In three mixed‐methods studies (Gobbi et al. 2018; McMahon et al. 2020; Stanish et al. 2019), qualitative analysis was utilised to analyse responses to structured interview questions. Of the nine intervention studies, only three were a randomised control trial (RCT; Fleming et al. 2023; Ptomey et al. 2024; Ptomey, Lee, et al. 2022b), and the remaining were quasi‐experiential (Bellamy et al. 2020; Pincus et al. 2019; Ryuh et al. 2022; Shih 2011; Shih et al. 2011; Sundahl et al. 2016). Most studies employed convenience or criterion sampling. Fourteen of the studies in the review examined individuals with Down syndrome (DS) exclusively. Studies used varying sources, such as parents, school officials/records and/or health care professionals, for reporting intellectual disability conditions for study inclusion criteria. Across studies, the levels of intellectual disability varied, including mild, moderate, and severe as reported by varying study sources (see Table 1). Eight studies in the review did not report the specific level of intellectual disability among study participants (Boddy et al. 2015; Carrogi‐Vianna et al. 2017; Downs et al. 2016; Fleming et al. 2023; Lobenius‐Palmér et al. 2018; McMahon et al. 2020; Stanish et al. 2019; Wang et al. 2022).

#### 3.1.3 Technology

##### 3.1.3.1 RQ 1: Types for Engagement in Physical Activity

Fifteen percent (*n* = 6) of all captured studies utilised exergaming technology to influence engagement in PA. In six studies, only two (McMahon et al. 2020; Ryuh et al. 2022) attempted to measure enjoyment and/or engagement with technology. Ryuh et al. (2022) utilised the Xbox 360 console to integrate interactions with a video dance game, as well as assessed enjoyment of PA, with the modified physical activity enjoyment scale (PACES) scale. McMahon et al. (2020) combined the usage of the VR exercise gaming platform VirZOOM and VR goggles to have participants accelerate the pedals of a stationary bike as a controller for VR games. For example, the faster the participant pedalled, the faster the vehicles in the games (e.g., bike, race car, horse, kayak) would go. McMahon et al. (2020) asked open‐ended questions to collect data from participants to understand their engagement and enjoyment with the immersive VR gaming experience. Two studies utilised the Nintendo Wii system platform, incorporating use of videogame playing (e.g., bowling, golf, dancing, tennis, baseball, and boxing; Carrogi‐Vianna et al. 2017; Pincus et al. 2019). Additionally, Shih (2011, 2011) used the Nintendo Wii Balance Boards and EeeBox minicomputer to evaluate performance of designated PA tasks.

Two studies implemented online technology resources, such as conferencing platforms, apps (e.g., Fitbit, Rooster money) uploaded to tablets, and exercise resources (Ptomey et al. 2024; Ptomey et al. 2022a). In one arm of a RCT, Ptomey et al. (2022a) delivered education and behavioural counselling sessions using Zoom video conferencing (via an iPad tablet) to assist participants in devising strategies to meet and engage in weekly PA goals (see Table 3). Only one study, Ptomey et al. (2024), examined the delivery of home‐based remote group exercise sessions using Zoom video conferencing via an iPad tablet. In the other study arm (Ptomey et al. 2024), the same home‐based exercise group intervention was delivered in addition to education/support sessions to both the adolescent and a parent (A + *P*) using Facetime video conferencing via an iPad tablet. Also, participants in both arms received intervention reminder messages and access to PA resources via iPad tablets (Ptomey et al. 2024). Furthermore, to influence engagement in PA, Ptomey et al. (2024) employed the use of NatWest Rooster Money (iPad app), so participants could earn virtual stars and exchange them for monetary incentives.

**TABLE 3:** Technology and physical activity in included studies (*N* = 45).

Author, year, country	Technology	Use of technology	PA	Results
Beets et al. (2007), US	Pedometer: WalkForLifeDuo	Measurement of PA	Walking 80‐m distance with pedometer placed in different areas	The location of the pedometer had minimal influence on accuracy of the step counts. Slow walking impacts the accuracy of step counts with this population.
Bellamy et al. (2020), Australia	Accelerometer: ActiGraph GT3X	Measurement of PA	Two 30‐min PA sessions/week	55% recruitment rate, 91% retention rate, 86% attendance rate, spent 38.4% of session in MVPA; increase in weight from mid intervention to 3‐month follow‐up.
Boddy et al. (2015), UK	Accelerometer: ActiGraph GT1M	Measurement of PA	Recess play	No differences in habitual PA, sedentary behaviour, or recess play behaviour between boys and girls. Spent their recess time alone or playing in small groups. None participated in large group plan; positive correlations were observed between alone time & PA.
Carrogi‐Vianna et al. (2017), Brazil	Accelerometer: Mma7361 triaxial accelerometer Nintendo Wii: Bowling and golf games	Measurement of PA Upper limb movement	15‐min bowling game, 5‐min break, 15‐min golf game	Accelerometry can be used to capture acceleration of movement during videogames. The results correspond to the current knowledge about DS, that is, that there is a general delay in development and a slowness in motor responses.
Downs et al. (2016), UK	Accelerometer: ActiGraph, Model GT1M	Measurement of PA	Monitored PA over 7 days	Mean habitual MVPA levels was 49.4 min/day, with only 23.7% (*n* = 9; 8 boys) of participants meeting the 60‐min/day MVPA guideline for health. Boys tended to engage in more PA than girls, and results demonstrate some large and potentially meaningful differences; for example, there was a 12‐min/day sex difference in MVPA. Boys accrued significantly more continuous LPA bouts and MPA bouts, than girls.
Einarsson et al. (2016), Iceland	Accelerometer: ActiGraph, model GT1M	Measurement of PA	Monitored PA over 7–10 days	TDI children were 44% more active and 24% less sedentary than children with ID during the weekday. An interaction in PA was observed between sex and group (*p* = 0.003). TDI boys were more active than TDI girls (*p* < 0.001). No difference between sex was found in the ID group (*p* = 0.583). TDI children were 25% more active during school hours and 73% more active after school than children with ID. Children with ID were more active during school than after school (*p* = 0.002).
Einarsson et al. (2015), Iceland	Accelerometer: ActiGraph, model GT1M	Measurement of PA	PA during wake hours for 7–10 consecutive day	ID participants were more sedentary and had less PA recorded than TDI group. This was seen for weekday and weekend data. Boys with ID were found to be more sedentary on weekends than weekdays (*p* = 0.018).
Esposito et al. (2012), US	Actical accelerometer	Measurement of PA	PA during wake hours over 7 days	Adolescents aged 14–15 years were the most sedentary and spent the least amount of time in light and moderate‐to‐vigorous physical activity. Physical activity decreases as DS children age, similar to that found among TDI. Participants spent a majority of their day engaged in sedentary activities. Most participants were not accumulating the recommended 60 min of moderate or vigorous PA.
Fleming et al. (2023), US	Accelerometer: ActiGraph wGT3X‐BT	Measurement of PA	Monitored PA daily over 6 months	During FBBI, mean (SE) MVPA increased by 4.1 (2.5) min/day and LPA by 24.2 (13.5) min/day. Mean (SE) difference in MVPA between participants in FBBI‐M and FBBI‐C at18 months was 14.0 (5.1) min/day (*p* = 0.005); mean (SE) difference in LPA was 47.4 (27.4) min/day (*p* = 0.08). Increasing PA through multicomponent behavioural intervention is possible in youth with ID.
Gobbi et al. (2018), Italy	Accelerometer: ActiGraph GT3X	Measurement of PA	An extracurricular peer‐tutored physical education	During PTPE, participants reported higher light intensity PA, enjoyment, and exertion than during SPE. Overweight participants showed less inactive time and higher light intensity PA during PTPE than during SPE.
Hao and Razman (2023), China	Accelerometer: ActiGraph wGT3X‐BT	Measurement of PA	Monitored PA during PE classes over 2‐week period	The mean time spent in MVPA during PE classes was 8.00 ± 2.10 min, accounting for only 22.88% of PE class time. As grade levels progressed, time spent in MVPA during PE classes tended to decrease; the fourth‐grade children tended to spend more time in MVPA during PE classes compared with the fifth and sixth grades (9.15 vs. 7.61 vs. 7.25 min, all *p* < 0.05). Boys spend significantly more time in MVPA during PE classes than girls; both in the entire sample and in each grade.
Izquierdo‐Gomez et al. (2017), Spain	Accelerometer: ActiGraph GT1M, GT3X, and GT3X+ models	Measurement of PA	Monitored PA during wake hours over 7 consecutive days	PA in adolescents with DS declined from baseline to follow‐ups. Youths who met PA guidelines at baseline demonstrated a greater decline in PA in 1‐ and 2‐year changes but they were also more likely to meet PA guidelines at 1‐ and 2‐year follow‐ups.
Izquierdo‐Gomez et al. (2015), Spain	Accelerometer: ActiGraph GT1M, GT3X, and GT3X+ models	Measurement of PA	Monitored PA during wake hours over 7 consecutive days	SES was negatively associated with total PA (*p* < 0.05). Participant age, SES and father PA were negatively associated with MVPA, while parental support and TV viewing time with friends were positively associated (all *p* < 0.05). Participant's age and father PA were also negatively associated with VPA, while TV viewing time with siblings were positively associated (all *p* < 0.05).
Lobenius‐Palmér et al. (2018), Sweden	Accelerometer: ActiGraph, Model GT1M	Measurement of PA	Monitored PA during wake hours over 7 consecutive days	Few youth with disabilities met PA recommendations, and they were less physically active and more sedentary than TDI youth. The hearing impairment and autism spectrum disorder groups were the most and least physically active, respectively. Older age and female sex were related to less PA and more sedentary time.
Matute‐Llorente et al. (2017), Spain	ActiTrainer uniaxial accelerometer	Measurement of PA	Monitored PA during wake hours over 7 consecutive days	Females with higher PA levels demonstrated increased integral (0.774 g/cm2 vs. 0.678 g/cm2) and superolateral femoral neck BMDs (0.696 g/cm2 vs. 0.595 g/cm2) compared to those with lower PA levels (*p* < 0.05).
Matute‐Llorente et al. (2013a), Spain	ActiTrainer uniaxial accelerometer	Measurement of PA	Walk on treadmill, increase speed until could not walk without running, then until exhaustion	Adolescents with DS spent less time in sedentary PA, MPA, VPA, and MVPA than those without DS.
Matute‐Llorente et al. (2013b), Spain	ActiTrainer uniaxial accelerometer	Measurement of PA	PA over 7 consecutive days	PA was lower in the DS group (*M* = 470.7, SD = 61.3) than in control group (*M* = 540.2, SD = 62.3); (*p* < 0.05). Age F (1, 29) = 43.45, (*p* < 0.05) and valid time F (1, 29) = 7.38, (*p* < 0.05) had a significant effect on LPA. None of adolescents with DS hit the minimum of 60 min of daily MVPA. Adolescents with DS who engage daily in more mins of total PA have higher BMD Z‐scores, mainly at the hip region.
McMahon et al. (2020), US	Virzoom exercise bike and the HTC VIVE VR googles Apple watch‐Smartwatches	Intervention tools to increase PA Measure HR	Stationary bike	All students increased the duration and intensity of all of their physical activity when using the VR exercise (exergaming) intervention.
Pan et al. (2015), Taiwan	Accelerometer: ActiGraph, Model GT1M	Measurement of PA	One PE class modified for students with ID and one general PE class	Adolescents with ID in self‐contained classrooms were less active during recess than the other two groups. They spent less percentage of time in MVPA during recess than did TDI.
Peiris et al. (2017), Australia	Accelerometer: SenseWear armband, RT3 monitor OxyCon mobile (measures gas exchange)	Measurement of PA Physiological measurement	Two 60‐min sessions, performed 1 week apart consisting of: sitting, standing, walking, running, and lying down	Both products were reliable for energy expended at rest. However, they were not valid during walking tasks.
Phillips and Holland (2011), UK	Accelerometer: ActiGraph, Model GT1M	Measurement of PA	Monitored PA during wake hours over 7 consecutive days	No individuals with intellectual disabilities met PA recommendations. Males were more active than females. PA declined and sedentary behaviour increased with age. Those with more severe levels of ID were more sedentary and less active, however any relationship was not significant when adjusted for confounding variables. Participants with DS engaged in significantly less PA than those with ID without DS and levels of activity declined significantly with age.
Pincus et al. (2019), US	Wii game system	Intervention tools to increase PA	Playtime (choice wii, motor toys, open space)	An exergaming condition produced the highest levels of activity. Two of 3 participants preferred the PA context to the sedentary. For the third participant, an intervention was included to increase activity. Although the intervention was successful, participant preference for the sedentary activity context remained unchanged.
Pitchford et al. (2018a), US	Accelerometer: Actigraph GT3X+	Measurement of PA	Monitored PA during wake hours over 7 consecutive days	DS participants had significantly higher BMI, total and abdominal body fat, and ratios of abdominal adiposity, as well as lower daily levels of MVPA than their TD peers.
Pitetti et al. (2009), US	Pedometer: Walk4Life Duo 2505 Video‐taped with Sony Mini Camcorder	Measurement of PA Video recording of intervention	Adapted PE class activity ex: indoor baseball, volleyball, basketball, field hockey, ball catching and throwing games, tag games	Pedometer registered steps were underestimated by 14% ± 16.5%. Pedometer registered time was overestimated by 8.7% ± 21.8%.
Ptomey et al. (2024), US	ActiGraph model wGT3x‐BT Fitbit Versa Charge 3 activity charger Phone (call, text, email) & Facetime Rooster Money iPad app Dropbox iPad app iPad tablet, pre‐loaded with the Fitbit app, Zoom video conferencing, SO & NCHPAD information for increasing PA loaded on iPad	Measurement of PA Reminders from health coaches Participant incentives Group video PA recorded sessions stored Graphic display of PA for health coaches AO and A + *P* arms receive orientation; A + *P* arm receives group video exercise sessions with health coach	Monitored PA over 18‐month period	Mixed modelling, controlling for baseline MVPA and season, indicated minimal but statistically significant changes in MVPA across 6 (*p* = 0.006), 12 (*p* < 0.001), and 18 months (*p* < 0.001). The change in MVPA in the two intervention arms did not differ significantly at any time point (all *p* > 0.05). Gross motor quotient and leg press strength improved significantly over time (*p* < 0.001), and these changes did not differ between intervention arms (all *p* > 0.05).
Ptomey et al. (2022a), US	ActiGraph model wGT3x‐BT tri‐axial accelerometer	Measurement of PA	Monitored PA for 7 days	Ninety‐two adolescents (15.5 ± 3.0 years old, 21.7% non‐White, 6.5% Hispanic, 56.5% female) provided valid accelerometer data. Average sedentary time was 494.6 ± 136.4 min/day and average MVPA was 19.8 ± 24.2 min/day. Age (*r* = 0.27, *p* = 0.01), diagnosis of congenital heart disease (r = −0.26, *p* = 0.01) and parent sedentary time (*r* = 0.30, *p* = 0.01) were correlated with sedentary time. BMI (r = −0.24, *p* = 0.03), waist circumference (r = −0.28, *p* = 0.01), identifying as White (r = −0.23, *p* = 0.03) and parent MVPA (*r* = 0.56, *p* < 0.001) were correlated with MVPA. After adjusting for the adolescent's age, sex, race, waist circumference, and total wear time, the association between parent and adolescent MVPA remained significant (b = 0.55, *p* < 0.01, partial η2 = 0.11).
Ptomey et al. (2022b), US	Fitbit Charge HRwireless activity tracker iPad tablet, which was pre‐loaded with the Fitbit app. Face to face arm: FTF arm were provided with a pedometer (Omron HJ‐320) ActiGraph model wGT3x‐BT tri‐axial accelerometer	Measurement of PA Graphic display of PA Measurement of PA Measurement of PA	PA and sedentary time were assessed at baseline and 6 months	No significant changes in LPA, MPA, MVPA, or sedentary time across the 6‐month intervention (all *p* > 0.05). No significant effects of group (p. 79), time (*p* = 0.10), or group‐by‐time interaction (*p* = 0.21) on changes in MVPA. Attendance at education counselling sessions (*r* = 0.26, *p* = 0.22) and frequency of self‐monitoring of MVPA were not significantly associated with change in MVPA (*r* = 0.26, *p* = 0.44). Baseline MVPA (*r* = 0.02, *p* = 0.92) and change in MVPA from baseline to 6 months (*r* = 0.13, *p* = 0.30) were not associated with changes in body weight.
Queralt et al. (2016), Spain	Pedometers: Yamax Digiwalker SW‐200	Measurement of PA	Monitored PA for 7 days	Significant differences in daily PA levels between boys and girls (12,630 and 9599 steps respectively, *p* < 0.05); Girls were less active than boys on weekdays (13,872 vs. 9868 steps; *p* = 0.016), during school time (7097 vs. 4802 steps, *p* = 0.005), and during recesses (1953 vs. 1147 steps; *p* = 0.033). Boys showed more PA on weekdays compared to weekends (13,872 vs. 10,188 steps; *p* = 0.015) and PA at school represented 50% of the participants' daily PA in both genders. There were no differences comparing weight status groups.
Ryuh et al. (2022), US	Xbox 360 console Kinect accessory	Intervention tools to increase PA	Just Dance 3 game	Findings indicated exergaming held an immediate impact on psychological and psychological responses in young adults with ID.
Shields et al. (2017a), Ireland	RT3 accelerometer	Measurement of PA	Monitored PA for 8 days	No significant correlation between PA and cardiovascular fitness or PA and body size. Children with DS who were fitter, had lower BMIs (*r* = −0.77, 95% CI −0.41 to −0.93) and smaller waist circumference (*r* = −0.75, 95% CI −0.36 to −0.92). Children with DS who had lower BMI and waist circumference had higher cardiovascular fitness.
Shields et al. (2017b), Australia	RT3 accelerometer	Measurement of PA	Monitored PA for 8 days	PA *M* = 441 counts per min (SD = 136). Time in at least moderate intensity activity was *M* = 64 min (SD = 32 min). Six participants met PA guidelines. No association between foot posture and PA, or the presence of foot deformity and PA. Only 20 of the participants had enough ActiGraph data. Footwear fit was negatively associated with PA (R2 change = 0.164, *p* = 0.03)
Shields et al. (2009), Australia	RT3 accelerometer	Measurement of PA	Monitored PA for 7 days	Only 8 children (42.1%) undertook at least 60 min of MVPA each day for 7 days. VPA per day was 22.9 min (SD = 13.1 min). None of the children performed 20 min of continuous VPA 3 times a week. Sedentary or light activity per day was 640.7 min (SD = 83.1 min). MVPA lasted 2.8 min (SD = 0.6 min), VPA lasted 2 min (SD = 0.6 min). Older children tended to record a lower level of PA.
Shih (2011), Taiwan	Nintendo Wii Balance Boards Eee Box minicomputer	Intervention tools to increase PA	Walking from one Wii board to the next	The two people with disabilities to significantly increased their target response (PA)—walking from one destination to another destination—to activate the control system to produce environmental stimulation during the intervention phases.
Shih et al. (2011), Taiwan	Nintendo Wii Balance Boards Eee Box minicomputer	Intervention tools to increase PA	Walking from one Wii board to the next	The Wii Balance Board can be extended from its default function, and turned into a high‐performance assistive device, in order to match the special needs of people with disabilities.
Sit et al. (2017), China	Accelerometr: ActiGraph GT3X model	Measurement of PA	Monitored PA for 3 days	Boys with mild ID, moderate ID, and SD problems were more physically active than boys with severe ID. No significant differences in MVPA across the disability types for girls. Girls with VI, mild ID, and severe ID were less sedentary than girls with severe ID. Children spent 70% of their day at school being sedentary and accrued little MVPA (*M* = 17 ± 4.2 min daily). Children with severe ID had especially low levels of MVPA. All three settings contributed to both MVPA and ST, with recess contributing more to MVPA than physical education or lunchtime.
Stanish et al. (2019), US	Actical accelerometers	Measurement of PA	Monitored PA for 7 days	Youth with ID spent less time in MVPA (33.5 vs. 46.5 min/day, *p* = 0.03) and were less likely to meet the US PA Guidelines than TD youth (6% vs. 29%, *p* = 0.01). Females with ID participated in PA more frequently than TD females (47.1 vs. 28.2 times/month, *p* = 0.008) and also reported engaging in a greater variety of PA (7.8 vs. 5.2 activities/year, *p* = 0.01). No differences between males in the frequency of PA participation or the number of activities performed. Both groups reported walking/hiking and active video as top activities. 84% of the participants with ID had enough ActiGraph data for analysis.
Sundahl et al. (2016), Sweden	Pedometer	Measurement of PA	Monitored PA for 5 consecutive days	Females without ID met recommendations regarding number of steps (10,000–12,000/day). No significant associations between total number of steps taken and BMI. Females with ID had a significantly lower (*p* = 0.005) mean total number of steps at 40,539 (SD = 18,257) compared to females without ID, 57,867 (SD = 22,021). Males with ID had a higher mean total number of steps at 49,590 (SD = 21,769), compared to 47,984 (SD = 18,657) for the males without ID, although this difference was not statistically significant (*p* = 0.776).
Wang et al. (2022), China	Accelerometer: ActiGraph wGT3‐BT	Measurement of PA	Monitored PA for 3 days	A significant positive correlation was found between MVPA and FMS, moderated by gender and age. For boys, object control skills were a significant predictor of MVPA time. For girls, locomotor skills were a significant predictor of MVPA time. For children with ID, object control skills were a significant predictor of MVPA time. Proficiency in FMS has a positive effect on increasing the level of MVPA in children & adolescents with ID. The locomotor skills scores remained significantly higher than the object control skills scores. For adolescents with ID, none of the skill variable were significant predictors of MVPA time.
Wouters et al. (2019), Netherlands	Accelerometer: Actigraph GT3x+	Measurement of PA	Monitored PA for 4 days	More than three quarters of the day were spent sedentary. The remaining time was spent with light intensity (8%, 53 ± 17 min), moderate intensity (9%, 59 ± 26 min) and vigorous intensity (5%, 33 ± 25 min); 47% of the participants were active enough to meet the recommendations of at least 60 min of MVPA every day. The number of steps per day was associated with boys (β = −0.33; *p* < 0.01) and having DS (β = −0.25; *p* < 0.05) in the first model. When motor development was added, sex and DS were no longer significant predictors, but motor development was. For cpm, adaptive behaviour became a significant predictor in the second model. Only 68 of the 130 participants had enough valid data to be included in the analysis.
Xanthopoulos et al. (2023), US	Accelerometer: SenseWear Mini armband, Professional v8.1	Measurement of PA	Monitored PA for 7 days	Youth with DS engage in more LPA and less SA than non‐DS youth. LPA was negatively associated with BMI‐Z and VFAT; this relationship persisted in adjusted models in both groups. PA interventions aimed at increasing LPA and decreasing SA, particularly in females, may be feasible and promising targets at improving adiposity and cardiometabolic risk in this population.
Xu and Wang (2023), China	Accelerometer: ActiGraph GT3X	Measurement of PA	Monitored PA	The proportions of participants who meet none, MVPA, ST (screen time), sleep duration and all three recommendations are 8.15%, 33.54%, 54.23%, 75.55%, and 17.55%, respectively. Older participants are less likely to meet the ST guidelines [OR: 0.931; 95% CI: 0.869–0.998] and more likely to meet the sleep guidelines (OR: 1.106; 95% CI: 1.016–1.204) than younger individuals. Participants with moderate ID are less likely to meet the sleep guidelines (OR: 0.345; 95% CI: 0.140–0.850) than those with profound ID.
Yang et al. (2024), South Korea	Fitbit Charge 5	Measurement of PA intensity	Monitored PA for 7 days	Sedentary time was higher for the adolescent group (*p* = 0.022), while LPA time was lower (*p* = 0.020). All measured PA patterns, excluding sedentary behaviour, decreased on weekends in both groups: steps (*p* = 0.002), LPA time (*p* = 0.028), and MVPA time (*p* = 0.004). Parental proxy questionnaires underestimated actual PA levels.
Yang et al. (2022), China	Accelerometer: GENEActiv	Measurement of PA	Monitored PA for 7 days	PA levels had positive dose–response associations with mental health, and the interaction between MVPA and SC was positively associated with social QoL. VPA had negative predictive effects on SC in children and adolescents with ID. Age, BMI, and school significantly moderated PA levels and QoL. Male participants with mild ID and higher parental education levels had higher levels of PA and QoL than their counterparts.
Zhong et al. (2025), China	Accelerometer: ActiGraph GT3X	Measurement of PA	Monitored PA for 7 days	Results indicated 43.97% of children and adolescents with ID presented with EBPs. After controlling for age, sex, ID severity and weight status the participants who meet the MVPA guideline exhibited significantly lower odds ratio for emotional symptoms (OR = 0.334, 95% CI [0.114–0.975], *p* = 0.045), peer problems (OR = 0.071, 95% CI [0.015–0.328], *p* < 0.001) and total difficulties (OR = 0.192, 95% CI [0.069–0.535], *p* = 0.002) compared with those who did not meet the guidelines.
Zhu et al. (2020), China	Accelerometer: ActiGraph wGT3‐BT	Measurement of PA	Monitored PA for 7 days	Findings from this study support the utilisation of a 1‐day familiarisation period, as well as discounting the final day of measurement, during data collection when examining physical activity behaviours among youth with moderate and severe ID.

*Note:* Adolescent only = AO; Adolescent + parent = AO + *P*; BMD = Bone mineral density; BMI = Body mass index; BMIZ = Body mass index z‐score; BPM = Beats per minute; CPM = Counts per minute; DS = Down syndrome; DXA = Dual‐energy x‐ray absorptiometry; EBPs = Emotional and behavioural problems; FBBI = family‐based weight‐loss behavioural intervention; FBBI‐M = maintenance intervention; FBBI‐C = control group; FMS = Fundamental movement skill; HR = Heart rate; ID = Intellectual disabilities; LPA = Light physical activity; MPA = Moderate physical activity; MVPA = moderate‐to‐vigorous‐intensity physical activity; NCHPAD = National Center on Health, Physical Activity and Disability; PA = Physical activity; PE = Physical education; PTPE = Peer‐tutored physical education; QOL = Quality of life; SA = Sedentary activity; SC = Self‐concept; SES = Socioeconomic status; SO = Special Olympics; SPE = School physical education; ST = Screen time; TD = Typical development; TDI = Typical developed individual; TV = Television; VFAT = Visceral fat; VI = Visual impairment; VPA = Vigorous physical activity; VR = Virtual reality.

##### 3.1.3.2 RQ 2: Monitoring and Measuring Physical Activity

The most common technology tools used in the studies to quantify and monitor PA behaviour patterns (e.g., steps, minutes) were accelerometers (*n* = 35) and pedometers (*n* = 5). Additionally, the intensity of PA measurement across studies was examined primarily with accelerometers with a range of models used (see Table 3). However, the data collection of participants' level of intensity in PA from the Pincus et al. (2019) study came from video‐recorded observations. Pincus et al. (2019) measured PA intensity with an adapted version of the activity codes from the Observational System for Recording Physical Activity in Children‐Home (OSRAC‐H) tool (McIver et al. 2009). Another study utilised a pictorial scale with verbal descriptors to report participants' objective estimation of exercise intensity (Ryuh et al. 2022). Three studies (Ptomey et al. 2024; Ptomey et al. 2022b; Yang et al. 2024) employed various models of Fitbit devices (see Table 3). The RCT study by Ptomey et al. (2022b) utilised the combination of a Fitbit, accelerometer, and pedometer to collect data about PA activity, including steps, heart rate, and sedentary time. McMahon et al. (2020) recorded participants' average heart rate during exercise sessions with a smartwatch (Apple Watch). Some studies (*n* = 3) examined the accuracy of pedometer and accelerometer utilisation among adolescents with ID (Beets et al. 2007; Peiris et al. 2017; Pitetti et al. 2009). Additionally, Peiris et al. (2017) utilised an OxyCon mobile device to measure energy expenditure among youths with DS to assess the reliability of two different accelerometer models. One study aimed to evaluate accelerometry use to capture acceleration and motor response of upper limb movement while participants were playing Wii bowling and golf video games (Carrogi‐Vianna et al. 2017).

##### 3.1.3.3 RQ 2: Psychometric Assessment of Technology Tools

The use of accelerometers and pedometers are the most common tools to quantify PA behaviour among people with intellectual disabilities (Pitchford et al. 2018b), as found in this review (see Table 3). Accelerometers offer many benefits to data collection, such as eliminating recall bias and capturing time‐stamped data and movement in finer detail than activity diaries. Unfortunately, variability in models of accelerometers, where participants wear the device on their bodies for sensitivity, and validated cut points to quantify activity intensity, differed across studies. Additionally, compliance protocols regarding individuals wearing accelerometers and pedometers vary widely by study. In this review, Beets et al. (2007) concluded that the accuracy of pedometer step counts are underestimated with slow walking among youth with intellectual disabilities, compounding validity and reliability issues (McGarty and Melville 2018). Furthermore, there is a potential for increased activity levels while wearing a device, potentially inducing reactivity in behaviour among participants across studies (Leung et al. 2017; Pitetti et al. 2009).

Additionally, differences in categorising PA intensity among different subgroups of individuals with intellectual disabilities is a concern in this area of research; researchers can sometimes apply technology tools with limited measurement evidence or feedback derived from individuals with intellectual disabilities (Pitchford et al. 2018b; Van Biesen et al. 2024). The Apple Watch and Fitbit devices were incorporated into four studies (McMahon et al. 2020; Ptomey et al. 2024; Ptomey, Lee, et al. 2022; Yang et al. 2024) in this review. Ptomey et al. (2024) and Yang et al. (2024) found that participants reported Fitbit devices were comfortable to wear, user‐friendly, and resembled a watch, which assisted with self‐assessment and self‐management of PA levels. One study reported the rate of refusal to wear the Fitbit device was only 12.5% (Yang et al. 2024). The use of qualitative methods to provide study participants' and their support networks' perspectives about compliance with technology tools could provide additional insights; however, no qualitative studies met the review criteria.

##### 3.1.3.4 RQ 3: Facilitators and Barriers of Technology

###### 3.1.3.4.1 Facilitators

Studies described adolescents with intellectual disabilities often found technology motivating, creating a play strategy incentive to perform PA (Carrogi‐Vianna et al. 2017; Pincus et al. 2019; Ptomey et al. 2024). Teachers reported that VR exercise bike games were fun for students and created a behaviour incentive for following directions in the classroom (McMahon et al. 2020). Other studies noted the use of a favourite video or song had a distracting effect during exercise, which increased PA duration and intensity (Ryuh et al. 2022; Shih 2011; Shih et al. 2011). Other studies suggested that commercial technology products were easy to use, portable, had good customer service technology support, and had modifications to default functions that could be adjusted for people with intellectual disabilities (Shih 2011; Shih et al. 2011; Yang et al. 2024). Additionally, studies suggested that with modelling, practice setting up game systems, and choice in game activities, adolescent participants followed instructions and vocal prompts to interact with the gaming technology with a high level of acceptance (Carrogi‐Vianna et al. 2017; McMahon et al. 2020; Pincus et al. 2019; Ptomey et al. 2024; Ryuh et al. 2022).

Other research indicated that video conferencing technology with an iPad provided a strategy for social interactions and collaborations between adolescent participants and the participants' health coaches (Fleming et al. 2023; Ptomey et al. 2024; Ptomey et al. 2022b). This type of technology approach can eliminate transportation challenges and provide opportunities for social support and community integration in a variety of settings. Additionally, real‐time data from the Fitbit wrist‐worn activity taker automatically transfers to a Fitbit app loaded on an iPad, which provides a graphic display. The display data includes daily steps, heart rate, minutes of sedentary time, time spent in light, and moderate and vigorous PA. Ptomey et al. (2022b, 2024) provided the graphic display data to participants and their parents for use in guiding and formulating bi‐weekly goal‐setting sessions with a health educator.

###### 3.1.3.4.2 Barriers

Studies described challenges with the use of technology to stimulate PA among adolescents with intellectual disabilities. First, adherence to wearing and complying with placement of accelerometers and pedometers to track PA were noted across studies. However, to assist with device use compliance, some studies implemented visual pictures within the verbal and written instructions for participants and families (Peiris et al. 2017; Xanthopoulos et al. 2023). In four studies, iPhone or iPad text messaging was sent to participants, parents, and/or guardians as reminders to wear and/or sync devices (Einarsson et al. 2015, 2016; Ptomey et al. 2024; Sundahl et al. 2016). Secondly, two studies identified potential cost barriers to exergaming accessibility as a facilitator of PA among adolescents with intellectual disabilities (McMahon et al. 2020; Ryuh et al. 2022). However, the price of this type of technology is now comparable to the price of “at‐home” exercise equipment; it is readily available to set up and removes transportation challenges for PA engagement. Thirdly, studies emphasised the importance of participants and their parents practicing and modelling game system setup, providing visual picture aids, and technical support for implementation. Another consideration with exergaming technology is the amount of screen time that participants use gaming systems to decrease risks of cybersickness and eye strain. Of the six studies included in this review that employed exergaming technology, only three specified participants' time spent utilising the systems (varied from 10 to 30 min per day; Carrogi‐Vianna et al. 2017; McMahon et al. 2020; Ryuh et al. 2022).

##### 3.1.3.5 RQ 4: Positive/Negative Impact of Technology Use for Physical Activity

###### 3.1.3.5.1 Engagement

The benefits of exergaming and VR gaming applications are that they can be considered an exercise modality that favours motivation, interest, and enjoyment to promote PA (Oakes 2022; Suárez‐Iglesias et al. 2021). In this review, studies found exergaming a play strategy method, distractor and an incentive for exercise (McMahon et al. 2020; Ryuh et al. 2022; Shih 2011; Shih et al. 2011). Three studies described outcomes utilising exergaming, including higher levels of PA (McMahon et al. 2020; Pincus et al. 2019); increased duration of PA and total calories burned (McMahon et al. 2020); increased HR; rate of perceived exertion (RPE); and enjoyment (Ryuh et al. 2022). Three studies found exergaming motivating to evaluate performance and movement during designated PA tasks for participants with intellectual disabilities (Carrogi‐Vianna et al. 2017; Shih 2011; Shih et al. 2011). In addition, studies that proposed exergaming applications allowed for choice in activities among adolescents with intellectual disabilities, creating a sense of self‐management (Carrogi‐Vianna et al. 2017; McMahon et al. 2020; Ryuh et al. 2022; Shih 2011; Shih et al. 2011).

Additionally, studies indicated that technology (e.g., exergaming, accelerometers, pedometers, smartwatches) can be incorporated into daily routines in school and home settings of adolescents with intellectual disabilities. Some studies suggested that exergaming and video conferencing technology assist with decreasing potential environmental barriers to PA, such as limited transportation options to exercise events and facilities (McMahon et al. 2020; Pincus et al. 2019; Ptomey, Lee, et al. 2022b; Ptomey et al. 2024; Ryuh et al. 2022). Virtual video conferencing also provided opportunities for social support and interaction for participation in fitness and exercise activities with peers, family and the community (Ptomey, Lee, et al. 2022b; Ptomey et al. 2024).

Some of the drawbacks to utilising technology for PA engagement, such as exergaming, can include cybersickness, nausea, eyestrain and dizziness (Kaimara et al. 2022). However, exergaming session length varied from 10 to 30 min across studies. Additionally, the use of an outcome measure for VR symptoms (e.g., the Virtual Reality Symptoms Questionnaire [VRSQ]), with demonstrated psychometric properties, was not utilised among the review studies to monitor symptoms.

## 4 Discussion

This scoping review depicted a novel approach for identifying and summarising literature to understand, not only technology utilisation for monitoring PA, but also facilitating engagement among adolescents with intellectual disabilities. Of the 45 studies that met inclusion criteria for this review, 80% were relatively recent (published between 2015 and 2025), revealing researchers' rising interest in PA of adolescents with intellectual disabilities in the past decade. Study designs varied in methodology, approach, and quality. Sixty‐nine percent of the included studies had a MMAT quality assessment score of 60% or less (i.e., met three or fewer criteria). Studies should address quality in design and reporting for replication and usability. Synthesis of the literature identified quantitative descriptive studies with convenience sampling were the most numerous. Studies lacked reports of race, ethnicity, and support needs, while 18% of studies did not report participants' specific level of intellectual disability. Thus, results cannot be generalised. With the cross‐sectional design among 73% of non‐intervention studies, causality cannot be indicated. While one study was longitudinal, more are needed to identify technology factors associated with PA. Of the quantitative studies, only 17% were intervention studies, including three RCTs. Evidence is still emerging, with most studies appearing to be non‐interventional; this suggests a significant need for additional research using experimental designs (McGarty and Melville 2018; Van Biesen et al. 2024).

### 4.1 Key Findings and Implications for Future Research

#### 4.1.1 Types of Technology for Engagement

Ten types of technology were found among the literature, including fitness trackers (e.g., Apple Watch, Fitbit Charge), iPads, minicomputers, accelerometers, pedometers, exergaming systems, and VR (see Table 2). However, only 15% (*n* = 6) of all captured studies utilised gaming technology (e.g., exergaming or VR) to influence engagement in PA. The majority of studies (*n* = 40) used technological devices to monitor and measure PA behaviour among adolescents with intellectual disabilities. According to Lieberman et al. (2011), McMahon and McMahon (2016), and Mesa‐Gresa et al. (2018), VR can be used in a variety of settings. It is also adaptable to age and physical and cognitive abilities. VR has shown preliminary promise in promoting PA in adolescents with intellectual disabilities. Its application for engaging adolescents through exergames has demonstrated a positive impact on physiological, social, emotional and behavioural health (Charlier et al. 2016; Lieberman et al. 2011; McMahon and McMahon 2016; Mesa‐Gresa et al. 2018; Qian et al. 2020). A multilevel perspective through a socioecological lens involves multiple influences on PA (i.e., intrapersonal, interpersonal, organisational, community and policy). Further exploration of VR within this lens is needed to understand accessibility and spontaneous, unplanned PA with family, peers and communities. Additionally, more exergaming technologies are connecting networks, linking players with each other for social play. Incorporating social support in studies featuring adolescents with intellectual disabilities and technology use should be explored in future research.

Furthermore, measurement of the concepts of “engagement” and/or “enjoyment” with technology use among adolescents with intellectual disabilities was limited. Only two studies attempted to capture formalised documentation (e.g., open‐ended questions, PACES) of feedback from adolescents with intellectual disabilities utilising technology. Additionally, measuring cybersickness among adolescents with intellectual disabilities for exergaming and VR applications was missing in the literature. This review identified that self‐reported measures capturing the subjective views of people with intellectual disabilities are needed to support inclusive and engaging research. Adapting research instruments (e.g., Likert‐type scales) with visual/pictorial aids for response alternatives among adolescents with intellectual disabilities is an immediate priority. In this way, adolescents can provide their own unique perspectives and understanding regarding their well‐being (Hartley and MacLean 2006; Pett et al. 2021). Given the health inequities faced by this population, it is essential to adapt and validate research measurement tools in consultation and collaboration with people with intellectual disabilities so that effective interventions can be designed, tested and implemented.

#### 4.1.2 Monitoring, Measuring, and Assessment

Technology (e.g., accelerometers and pedometers) were utilised in 89% of studies to quantify and monitor PA behaviour patterns (e.g., steps, minutes, intensity) among adolescents with intellectual disabilities. However, different models wear protocols, and compliance with devices vary across studies. This makes comparisons across participant groups challenging, which is consistent with existing literature (Pitchford et al. 2018b). Furthermore, some studies incorporated video recordings to capture and code PA behaviour, while other studies utilised fitness trackers and smartwatches to collect data. Research exploring the use of smartwatches (e.g., Apple Watches) to measure heart rate indicates a high degree of accuracy (Wallen et al. 2016). El‐Amrawy and Nounou (2015) compared several popular fitness trackers and found the Apple Watch was one of the most accurate tested for heart rate. However, the measurement evidence from this study was derived from typically developing adults and may not generalise to samples including adolescents with intellectual disabilities. Future studies providing validity and reliability evidence for measuring PA behaviour with commercial activity trackers among adolescents with intellectual disabilities would strengthen research efforts in this area.

#### 4.1.3 Facilitators and Barriers

This review identified important factors related to technology use associated with PA engagement. The studies featured in the scoping review suggest adolescents with intellectual disabilities found technology motivating. Interventions created a play space of choice and distraction, while also generating incentive to engage in PA. Moreover, literature advocated for incorporating commercial technology products into research that were easy to use, portable, and had varied modification features that could be adjusted for adolescents with intellectual disabilities. Consequently, studies supported integrating modelling techniques into research protocols to assist with practice and use of technology (especially gaming technology) to enhance acceptance and understanding among adolescents with intellectual disabilities, their families and support personnel.

Additionally, studies in the review discussed video conferencing technology, which provides a venue for increased social interactions, community integration and collaborations among adolescents with intellectual disabilities, highlighting a multilevel approach to PA from a socioecological perspective. This technology decreased environmental barriers, such as transportation and exercise accessibility issues. Technology (e.g., exergaming and VR) allows for accessible, spontaneous and unplanned PA with family and peers. This can help to provide social motivation, which is a strong facilitator of PA among adolescents with intellectual disabilities (McGarty and Melville 2018; Shields et al. 2012).

Other literature in this review identified challenges with the adherence of wearing measurement devices, such as accelerometers, pedometers and fitness trackers. However, some researchers in the review implemented strategies in their research protocols to assist with these issues. These strategies included utilising visual picture aides or picture cards to display a symbolised meaning, in addition to modelling along with written and verbal instructions.

#### 4.1.4 Positive Impact

A major finding among the studies includes evidence that exergaming and VR applications can be considered a viable modality for PA among adolescents with intellectual disabilities. Studies suggest exergaming was not only a play strategy method, distractor and motivator for exercise, but also increased duration of PA, rate of perceived exertion, HR, calories burned and enjoyment. In some studies, exergaming activities allowed adolescents a mechanism of choice in a variety of gaming options to create a sense of self‐management in exercise activities. Some studies incorporated technology into daily routines in home and school settings. Thus, to impact health outcomes, multilevel interventions across a socioecological framework must explore the potential for interprofessional collaborations in broader settings to engage adolescents with intellectual disabilities in PA. Findings from this review provide insights into gaps in the literature for future research.

### 4.2 Limitations and Strengths of the Scoping Review

It is important to acknowledge the limitations of this scoping review, despite adherence to the PRISMA‐ScR checklist (Tricco et al. 2018). First, only 45 studies were included. It is possible that some studies were not reached within the literature using key search terms. Second, we excluded grey literature and studies not published in English. Additionally, this review is predominantly quantitative and lacks qualitative studies. This may be a perceived limitation of our methodological framework, but it also marks an area for integrating qualitative approaches into future research. Third, a majority of the research was conducted in the United States, which limits variability related to culture, race and nationality diversity within the findings. Finally, the results of this review may be limited to adolescents with intellectual disabilities who have differing verbal, adaptive behaviour and cognitive abilities, potentially limiting the generalizability and applicability of the findings. Further research is needed among adolescents with intellectual disabilities to differentiate how technology is experienced.

However, our review has several strengths. A key strength includes the utilisation of Arksey and O'Malley's (2005) comprehensive methodological framework, which allowed us to yield an extensive quantity of relevant literature, review diverse study designs, identify gaps in research, and provide directions for future study. Further, we pre‐registered the scoping review in OSF to promote transparency and help avoid duplication of research efforts. We also utilised the MMAT to review and appraise the quality of studies in a meaningful way, which is often an optional step in scoping reviews. Finally, we included adolescents aged 10–21 years with intellectual disability conditions across levels (e.g., mild, moderate, severe), which includes transition‐aged youth eligible for special education services under IDEA Part B (US Department of Education 2023).

## 5 Conclusion

This scoping review identified and summarised research about the types of technology that have been utilised and assessed (e.g., barriers/facilitators) among adolescents with intellectual disabilities; it also evaluated what is known about factors related to technology use associated with PA engagement in this population. Future research should address key gaps by exploring the use of exergaming, measuring adolescent PA engagement and incorporating perspectives of adolescents with intellectual disabilities in the development, design, testing and implementation of future interventions and measurement tools. Standardised methods are needed with high intervention fidelity so studies can be compared. Research examining validity and reliability for measuring PA behaviour with commercial technology devices among adolescents with intellectual disabilities is needed. Also, facilitators of technology use outweigh barriers for use, so advancing scientific rigour within a socioecological lens with more qualitative, longitudinal, and intervention studies is vital. Lastly, from information captured in this review, future work is essential to increase PA and engagement among adolescents with intellectual disabilities to address chronic conditions and health disparities.

## Author Contributions

**Patricia West:** conceptualization; methodology; software; validation; analysis; investigation; resources; visualisation; supervision; project administration; writing – original draft; writing: drafting and revising. **Karla Palmer:** analysis; resources; validation; visualisation; writing – review and editing. **Brian Abery:** conceptualization; methodology; supervision; validation; visualisation; writing – review and editing. **Jessica Sender:** software; resources; visualisation; writing – review and editing. **Alli Walsh:** analysis; resources; validation; visualisation; writing – review and editing. **Gwen Wyatt:** conceptualization; methodology; supervision; visualisation; writing – review and editing.

## Funding

The authors have nothing to report.

## Ethics Statement

The authors have nothing to report.

## Consent

The authors have nothing to report.

## Conflicts of Interest

The authors declare no conflicts of interest.

## Data Availability

Data sharing not applicable – no new data generated in this review.

## Appendix A

### CINAHL

((intellectual* OR development* OR cognitive*) AND (disabl* OR disabil* OR delay*) OR autis* OR “autistic disorder” OR “autism spectrum disorder” OR ASD OR “down syndrome” OR (MH “Intellectual Disability”) OR (MH “Developmental Disabilities”) OR (MH “Autistic Disorder”) OR (MH “Down Syndrome”) OR “trisomy 21”) AND (Adolesc* OR transit* OR teen* OR “young adult” OR (MH “Adolescence”) OR (MH “Young Adult”) OR (MH “Transition to Adulthood”)) AND (“physical activit*” OR exercis* OR fitness OR “physical fitness” OR (MH “Physical Activity”) OR (MH “Physical Fitness”) OR (MH “Exercise”)) AND (“fitness tracker*” OR “activity tracker*” OR smartwatch OR fitbit OR garmin OR “apple watch” OR “wearable device*” OR “virtual realit*” OR “augmented realit*” OR exergam* OR “video game*” OR videogam* OR games OR game OR gaming Or (MH “Fitness Trackers”) OR (MH “Virtual Reality”) OR (MH “Augmented Reality”) OR (MH “Video Games”) OR (MH “Exergames”) OR pedometer* OR acceleromet* Or (MH “Pedometers”) OR (MH “Accelerometers”))

#### PubMed

((((Intellectual OR development* OR cognitive*) AND (disabl* OR disabil* OR delay*) OR autis* OR “down syndrome” OR “autistic disorder” OR “autism spectrum disorder” OR ASD OR “Autistic Disorder”[Mesh] OR “Autism Spectrum Disorder”[Mesh] OR “Down Syndrome”[Mesh] OR “trisomy 21”) AND (Adolesc* OR transit* OR teen* OR “young adult” OR “Adolescent”[Mesh] OR “Young Adult”[Mesh])) AND (“physical activity” OR “physical activities” OR “Exercise”[Mesh] OR exercis* OR fitness OR “physical fitness” OR “Physical Fitness”[Mesh])) AND (“fitness tracker” OR “fitness trackers” OR “Fitness Trackers”[Mesh] OR “activity tracker” OR “activity trackers” OR smartwatch OR fitbit OR garmin OR “apple watch” OR “wearable device” OR “wearable devices” OR “virtual reality” OR “virtual realities” OR “augmented reality” OR “augmented realities” OR exergam* OR “video game” OR videogame* OR games OR game OR gaming OR “Virtual Reality”[Mesh] OR “Exergaming”[Mesh] OR pedometer OR acceleromet*)

#### Embase

((intellectual* OR development* OR cognitive*) AND (disabl* OR disabil* OR delay*) OR autis* OR ‘down syndrome’/exp. OR ‘down syndrome’ OR ‘autism spectrum disorder’/exp. OR ‘autism spectrum disorder’ OR ‘asd’/exp. OR asd OR ‘autistic disorder’/exp. OR ‘autistic disorder’ OR ‘trisomy 21’/exp. OR ‘trisomy 21’) AND (adolescen* OR transit* OR teen* OR ‘young adult’/exp. OR ‘young adult’) AND (‘physical activit*’ OR exercis* OR ‘fitness’/exp. OR fitness OR ‘physical fitness’/exp. OR ‘physical fitness’) AND (‘fitness tracker*’ OR ‘activity tracker*’ OR ‘smartwatch’/exp. OR smartwatch OR ‘fitbit’/exp. OR fitbit OR garmin OR ‘apple watch’/exp. OR ‘apple watch’ OR ‘wearable device*’ OR ‘virtual realit*’ OR ‘augmented realit*’ OR exergam* OR ‘video game*’ OR games OR ‘game’/exp. OR game OR gaming OR pedometer* OR acceleromet*)

#### PsycINFO

noft((intellectual* OR development* OR cognitive*) AND (disabl* OR disabil* OR delay*) OR autis* OR “down syndrome” OR “autism spectrum disorder” OR ASD OR “autistic disorder” OR “trisomy 21”) AND noft(Adolescen* OR transit* OR teen* OR “young adult”) AND noft(“physical activit*” OR exercis* OR fitness OR “physical fitness”) AND noft(“fitness tracker*” OR “activity tracker*” OR smartwatch OR fitbit OR garmin OR “apple watch” OR “wearable device*” OR “virtual realit*” OR “augmented realit*” OR exergam* OR “video game*” OR games OR game OR gaming OR pedometer* OR acceleromet*).

#### SportDiscus

((intellectual* OR development* OR cognitive*) AND (disabl* OR disabil* OR delay*) OR autis* OR “down syndrome” OR “autism spectrum disorder” OR ASD OR “autistic disorder” OR “trisomy 21”) AND (Adolescen* OR transit* OR teen* OR “young adult”) AND (“physical activit*” OR exercis* OR fitness OR “physical fitness”) AND (“fitness tracker*” OR “activity tracker*” OR smartwatch OR fitbit OR garmin OR “apple watch” OR “wearable device*” OR “virtual realit*” OR “augmented realit*” OR exergam* OR “video game*” OR games OR game OR gaming OR pedometer* OR acceleromet*)
